# Beneficial Effect of Short-Term Supplementation of High Dose of Vitamin D_3_ in Hospitalized Patients With COVID-19: A Multicenter, Single-Blinded, Prospective Randomized Pilot Clinical Trial

**DOI:** 10.3389/fphar.2022.863587

**Published:** 2022-07-04

**Authors:** Miguel Cervero, Daniel López-Wolf, Guiomar Casado, Maria Novella-Mena, Pablo Ryan-Murua, María Luisa Taboada-Martínez, Sara Rodríguez-Mora, Lorena Vigón, Mayte Coiras, Montserrat Torres

**Affiliations:** ^1^ Internal Medicine Service, Hospital Universitario Severo Ochoa, Leganés, Spain; ^2^ Internal Medicine Service, Hospital Universitario Fundación Alcorcón, Alcorcón, Spain; ^3^ Immunopathology Unit, National Center of Microbiology, Instituto de Salud Carlos III, Madrid, Spain; ^4^ Internal Medicine Service, Hospital Universitario Príncipe de Asturias, Madrid, Spain; ^5^ Internal Medicine Service, Hospital Universitario Infanta Leonor, Madrid, Spain; ^6^ Internal Medicine Service, Hospital Universitario de Cabueñes, Gijón, Spain

**Keywords:** COVID-19, SARS-CoV-2, vitamin D_3_ supplementation, biochemical parameters, risk factors

## Abstract

There is now sufficient evidence to support that vitamin D deficiency may predispose to SARS-CoV-2 infection and increase COVID-19 severity and mortality. It has been suggested that vitamin D_3_ supplementation may be used prophylactically as an affordable and safe strategy that could be added to the existing COVID-19 standard treatment. This multicenter, single-blinded, prospective randomized pilot clinical trial aimed to evaluate the safety, tolerability, and effectiveness of 10,000 IU/day in comparison with 2000 IU/day of cholecalciferol supplementation for 14 days to reduce the duration and severity of COVID-19 in 85 hospitalized individuals. The median age of the participants was 65 years (Interquartile range (IQR): 53–74), most of them (71%) were men and the mean baseline of 25-hydroxyvitamin D (25(OH)D) in serum was 15 ng/ml (standard deviation (SD):6). After 14 days of supplementation, serum 25(OH)D levels were significantly increased in the group who received 10,000IU/day (*p* < 0.0001) (*n* = 44) in comparison with the 2,000IU/day group (*n* = 41), especially in overweight and obese participants, and the higher dose was well tolerated. A fraction of the individuals in our cohort (10/85) developed acute respiratory distress syndrome (ARDS). The median length of hospital stay in these patients with ARDS was significantly different in the participants assigned to the 10,000IU/day group (*n* = 4; 7 days; IQR: 4–13) and the 2,000IU/day group (*n* = 6; 27 days; IQR: 12–45) (*p* = 0.04). Moreover, the inspired oxygen fraction was reduced 7.6-fold in the high dose group (*p* = 0.049). In terms of blood parameters, we did not identify overall significant improvements, although the platelet count showed a modest but significant difference in those patients who were supplemented with the higher dose (*p* = 0.0492). In conclusion, the administration of 10,000IU/day of vitamin D_3_ for 14 days in association with the standard clinical care during hospitalization for COVID-19 was safe, tolerable, and beneficial, thereby helping to improve the prognosis during the recovery process.

## Introduction

Severe acute respiratory syndrome coronavirus 2 (SARS-CoV-2) is an enveloped positive-sense RNA betacoronavirus that appeared in Wuhan (China) in December 2019 and is the cause of coronavirus disease 2019 (COVID-19). COVID-19 has a variable clinical presentation from asymptomatic to milder symptoms or, far more seriously, as a severe and often deadly respiratory disease (Chen et al., 2020). The initial symptoms of COVID-19 mainly include fever, cough, myalgia, or fatigue and some patients develop gastrointestinal problems, such as diarrhoea and vomiting. In the later stages of the disease may occur dyspnea, hypoxia, confusion, or chest pain and gradually progress into complications such as acute respiratory distress syndrome (ARDS), which may result in lung injury, decreased oxygen saturation, and widespread tissue damage, thus, causing a multiple organ system failure, unfavourable prognosis, and even death (Hu et al., 2021, 2020).

The main cause of COVID-19 disease severity and death in patients is the extreme inflammatory response caused when the damaged cells trigger lung inflammation, largely mediated by proinflammatory macrophages and granulocytes, along with severe lymphopenia, neutrophil activation, thrombosis, and massive mononuclear cell infiltration in multiple organs (Merad and Martin, 2020). Accordingly, severe or fatal cases of COVID-19 disease are associated with elevated levels of interleukin-6 (IL-6), IL-7 and reduced levels of cytokines related to T-cell antiviral activity, such as IL-2, IL-12 or interferon (IFN)-γ (Vigón et al., 2021), as well as other laboratory abnormalities that include elevated levels of urea, creatinine, tissue markers (adipose tissue, kidney and muscle), ferritin, C-reactive protein (CRP), lower counts of lymphocytes (<1,000/µL), and platelets (<100 × 10^9^/L), as well as reduced levels of albumin (Goyal et al., 2020; Malik et al., 2021).

For a long time, no evidence-based or clinically demonstrated strategy was shown for the treatment of COVID-19 (Mishra and Tripathi, 2021). Different drugs, designed for treating other diseases, have been used to limit the adverse effects of viral pathogenesis, such as antivirals (e.g., remdesivir), IL-6 inhibitors (e.g., tocilizumab), Janus kinase (JAK) inhibitors (e.g., baricitinib), or corticosteroids (e.g., dexamethasone, methylprednisolone). The excessive immune response and inflammation in severe COVID-19 patients have prompted many clinical studies to use a combination of different drugs (Esposito et al., 2020). As a consequence, FDA recently authorized the simultaneous administration of nirmatrelvir and ritonavir for the treatment of mild-to-moderate COVID-19 in children and adults, as well as oral molnupiravir for adults, but this authorization has only been issued in case of emergency for those individuals with high risk to progress to severe disease (Couzin-Frankel, 2021; Parums, 2022).

Risk factors such as older age, cardiovascular disease, diabetes mellitus, chronic respiratory disease, arterial hypertension, and cancer are associated with a higher probability to develop a severe form of COVID-19 (Huang et al., 2020; Jordan et al., 2020; Yang et al., 2020). In addition, there is now sufficient evidence to indicate that pre-existing conditions such as vitamin D deficiency may also predispose to SARS-CoV-2 infection and increase COVID-19 severity (Bergman, 2021; Rhodes et al., 2021; Seal et al., 2022). Vitamin D is widely known for its role in calcium and phosphate balance but supports also key functions in many organs, including the brain, muscle and the immune system (Carlberg and Muñoz, 2022; Holick, 2022). Its immunomodulatory role promotes the development of an anti-inflammatory and antiviral environment and consequently, its deficiency seems to contribute to airway and gastrointestinal infectious illnesses (Ilie et al., 2020; Watkins, 2020). It has been suggested that vitamin D_3_ supplementation may be used prophylactically as an affordable and safe treatment strategy that could be added to existing COVID-19 protocols. However, there is still no clear evidence that vitamin D_3_ supplementation can prevent the severity and/or mortality of COVID-19.

Accordingly, the main objective of this study was to evaluate the safety, tolerability and effectiveness of vitamin D_3_ supplementation to reduce the duration and severity of COVID-19 in hospitalized individuals who were recruited for a randomized pilot clinical trial to receive the moderate dose of 2000 International Units (IU)/daily of cholecalciferol or a higher dose of 10,000 IU/daily for 14 days, in combination with the standard drug therapeutic regimen.

## Methods

### Study Design

This is a multicenter, single-blinded, prospective randomized pilot clinical trial (RCT) to evaluate the safety, tolerability, and effectiveness of the administration of daily oral supplementation of a high dose of cholecalciferol (vitamin D_3_) (10,000 IU/day) in comparison with a moderate dose of cholecalciferol (2000 IU/day) and in combination with the standard drug regimen therapeutic. Due to the potential effectiveness of vitamin D_3_ supplementation, and following the ethical tenet of beneficence, this pilot clinical trial did not include a placebo arm. The moderate (2000 IU/day) dose was selected following the Endocrine Society Practice Guidelines on Vitamin D which recommends the intake of 1,500–2,000 IU/day of vitamin D_2_ or D_3_ for adults over 18 year-old to achieve an optimal blood concentration of 25(OH)D in the range of 75–100 nmol/L (30–40 ng/ml) (Holick et al., 2011). The high dose (10,000 IU) was selected because the amount of vitamin D_3_ made in the skin after Sun exposure has been estimated within a range from 10,000 to 25,000 IU/day (Stamp et al., 1977; Holick and Chen, 2008; McCullough et al., 2019) and also because the daily intake of 10,000 IU/day for 5 months has been considered safe in a healthy population, without reporting events of hypercalcaemia or an increase in the urinary calcium excretion (Heaney et al., 2003; Holick and Chen, 2008). This study was sponsored by Hospital Universitario Severo Ochoa (Madrid, Spain) and was conducted between June 2020 and March 2021 in this hospital and other four Tertiary Care Hospitals located in Spain: Hospital Universitario Infanta Leonor (Madrid), Hospital Universitario Fundación Alcorcón (Alcorcón, Madrid), Hospital Universitario Príncipe de Asturias (Alcalá de Henares, Madrid), and Hospital Universitario de Cabueñes (Gijón, Asturias).

### Participants

The eligible participants were patients aged 18 years or older diagnosed with COVID-19 pneumonia based on clinical-radiological criteria and positive for SARS-CoV-2 infection confirmed by laboratory detection (RT-PCR), with values of oxygen saturation <94% and levels of 25(OH)D in serum <30 ng/ml. The exclusion criteria were pregnancy or to be lactating, patients who were participating in other clinical trials with drugs with potential antiviral action for COVID-19, on treatment with digoxin, with evidence of Multiple Organ Dysfunction Syndrome (MODS), or who were requiring mechanical ventilation at the time of inclusion. Patients who had hypersensitivity to cholecalciferol or the excipient refined olive oil, had hypercalcemia or hypercalciuria, were diagnosed with hereditary fructose intolerance, sarcoidosis or hyperparathyroidism, had glucose-galactose malabsorption, sucrose insufficiency or chronic kidney disease (stage 4; estimated glomerular filtration rate (eGFR) < 30), or who were expected to be transferred to another hospital in the following 96 h or to die in the next 24–48 h were also excluded.

### Ethical Considerations

This pilot clinical trial (protocol ID 01052020) was previously reviewed and approved by the Spanish Agency for Medicines and Health Products (AEMPS) (EudraCT Number 2020–002312–43) and by the Drug Research Ethics Committee of the Hospital Universitario Severo Ochoa (Madrid, Spain) (https://reec.aemps.es/reec/estudio/2020-002312-43). The study was conducted under the recommendations for Clinical Studies and Drug Evaluation in Humans, contained in the latest version of the Declaration of Helsinki, and the Spanish and European Legislation on Clinical Studies (Real Decreto 1090/2015; European Regulation 536/2014). Written or oral informed consent was obtained from all patients.

### Procedures

Following eligibility screening (visit 1), patients were randomized according to a computer-generated process by blocks series sizes of 5 (STATA 14.2 software; StataCorp LLC, College Station, TX) to receive 10,000 IU or 2000 IU of cholecalciferol (1:1) once daily for 14 days. Study medication was presented as an oral solution containing 25,000 IU/2.5 ml of cholecalciferol (Grupo Italfarmaco, Madrid, Spain) (National Code 7188605; ATC code A11CC05). This medication was provided by the pharmacy of each hospital, where the batches of the vials were registered, and administrated by a nurse according to the medical prescription. Throughout the study, all patients received any concomitant medication deemed necessary to provide adequate standard care, according to local recommendations. On-treatment, the visits occurred at seven (visit 2, day 7) and fourteen (visit 3, day 14) days. After 14 days of the end of the treatment, a safety follow-up visit (visit 4, day 28) occurred. At baseline and each visit, patients underwent a physical examination (including vital signs), a chest X-ray or imaging test, an electrocardiogram, and a blood sample collection. Data of the study were collected and recorded using the Research Electronic Data Capture tool (REDCap) online platform (https://e-clinicos.com/redcap/; Vanderbilt University, Nashville, TN), including the following data: age, sex, ethnicity, body mass index (BMI), main coexisting comorbidities (chronic heart/pulmonary/inflammatory/kidney disease, arterial hypertension, asthma, cirrhosis, neoplasia, HIV-1 infection/AIDS, obesity, dementia, malnutrition, smoker), respiratory status (respiratory rate, oxygen flow, ventilatory support), radiological score (number of affected zones of lung parenchyma), vital signs (body temperature, heart rate, breath rate, systolic blood pressure, diastolic blood pressure), symptoms (fever, malaise, upper respiratory tract symptoms, dyspnea, chest pain, cough, expectoration, hemoptysis, myalgia, headache, confusion, seizures, abdominal pain, nausea, vomiting, diarrhea, rash, anosmia), biochemical parameters (leucocytes, neutrophils and lymphocytes count, haemoglobin, platelets, D-Dimer, aPTT, fibrinogen, glucose, creatinine kinase, sodium, potassium, albumin, bilirubin, alanine aminotransferase (ALT/GPT), aspartate aminotransferase (AST/GOT), C-reactive protein (CRP), lactate dehydrogenase (LDH), procalcitonin, ferritin, calcium, phosphate, iPTH and vitamin D), treatments administered during admission (corticosteroids, tocilizumab, remdesivir, ceftriaxone, azithromycin, heparin), together with their previous or indicated treatments. Data were also collected on safety, adverse effects, symptom onset, length of hospital stay (LOS), the occurrence of ARDS, defined by the ratio arterial oxygen pressure/inspired oxygen fraction (PaO_2_/FiO_2_) <300 mm Hg, the admission at the Intensive Care Unit (ICU), and mortality.

### Vitamin D Quantification

The quantification of the level of 25(OH)D (ng/ml) in serum samples was performed with the automated immunoassays Liaison 25(OH) Vitamin D Total assay DiaSorin Liaison XL (DiaSorin, Italy) in the participating hospitals, according to the manufacturer’s instructions.

### Performance Measures

The primary outcome measure was to reach a serum 25(OH)D concentration ≥30 ng/ml (75 nmol/L) after 14 days of supplementation without the presence of any adverse effects. The secondary outcomes were the LOS, defined as the days that participants remained hospitalized from admission until the hospital discharge or death, as well as the development and evolution of blood biomarkers of inflammation. Other pre-specified clinical outcomes were the need for high-flow oxygen, ICU admission and mortality. The criteria for patient discharge were no requirement for supplemental oxygen, absence of fever, and oxygen saturation >94%.

### Safety Assessment

The safety assessment was carried out according to the scheme that included a detailed medical history, a physical examination and a biochemical and blood examination at each visit. Any adverse event that occurred during the study was recorded.

### Statistical Analysis

The primary analyses used the intention to treat (ITT) analysis set. The data were evaluated for normality using the Kolmogorov-Smirnov test, and mean ± SD was calculated to represent data following a normal Gaussian pattern, and median and interquartile range for skewed data. Categorical data were expressed as percentages. Group comparisons were done using Mann-Whitney U-test, t-student test or Wilcoxon signed-rank test for numerical data and Chi-square (χ2) or Fisher’s exact test for categorical data. Predictors related to the LOS were identified using multiple linear regression analysis. Predictors related to the ARDS were identified using multiple logistic regression analysis. A two-factor mixed factorial split-plot design was used, with an intersubject factor (2000 IU/day vs. 10,000 IU/day) and an intrasubject factor (baseline, visit 2, and visit 3) using a mixed ANOVA and multiple comparisons between the three-time periods (Repeated Measures Analysis with Stata, n. d.). The statistical analyses and graphics were performed using STATA 14.2 software. *p*-values lower than 0.05 (two-tailed) were considered to be statistically significant.

## Results

### Participants

**FIGURE 1:**
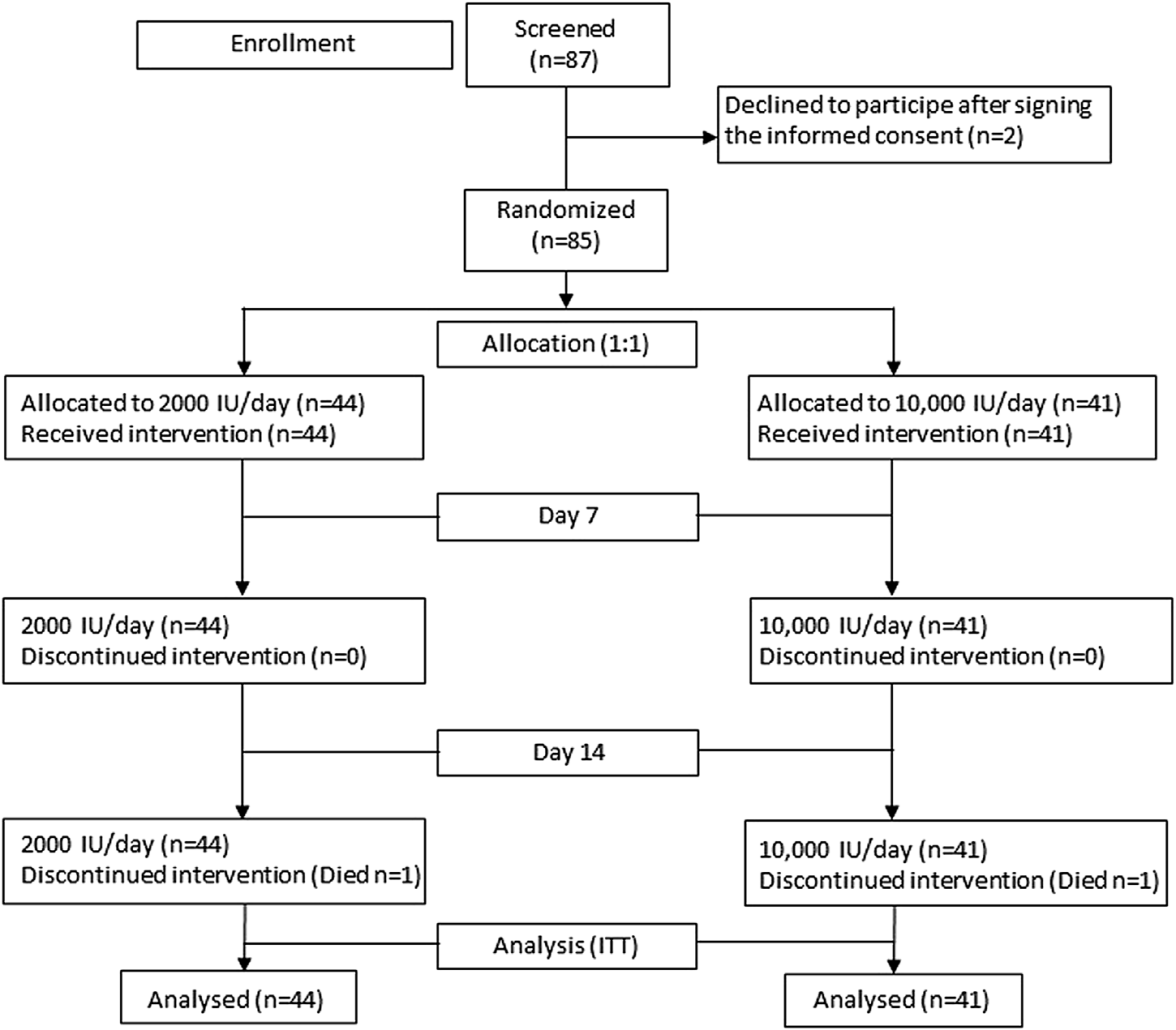
Flowchart of the study design. ITT: intention to treat.

A total of 87 participants were enrolled in the study. Of them, two participants that were randomized to the 10,000 IU/day group refused to participate and withdrew consent without having taken the study medication. Finally, 44 participants were assigned to take 2000 IU/day (moderate dose) of cholecalciferol and 41 participants 10,000 IU/day (high dose) of cholecalciferol (Figure 1). A summary of the characteristics of the participants assigned to each treatment group is shown in Table 1. The median time from the onset of the symptoms before admission was 7 days (IQR: 6–10). Median age of the participants was 65 years (IQR: 53–74), 71% of them were male and 54% were obese. The main conditions were hypertension (48%), dyslipidaemia (36%), and diabetes (22%), and at least one of these coexisting diseases were described in 34% of the participants. Most of the participants (86%) showed bilateral pneumonia at chest x-rays. According to the ventilatory support, 85% of the participants received oxygen with nasal goggles (between 1 and 5 L/min) and 15% of them with reservoir (between 6 and 15 L/min). Mean serum 25(OH)D basal levels were 14.8 ng/ml (SD: 6.2). The treatments administered included corticosteroids such as dexamethasone and methylprednisolone (87%), ceftriaxone (59%), azithromycin (44%), IL-6 antagonists such as tocilizumab (25%), and remdesivir (15%). There were no differences between groups at baseline regarding the demographic characteristics, symptoms, vital signs, blood biochemistry data, or treatments, although the level of haemoglobin was significantly higher (*p* = 0.006) in the group of participants assigned to the 10,000 IU/day group (Supplementary Table S1).

**TABLE 1:** Baseline clinical and biochemical characteristics in hospitalized patients with COVID-19 who received 2000 IU/day and 10,000IU/day of cholecalciferol.

Characteristics	Overall	Treatment Group	
	(*n* = 85)	2000 UI/day (*n* = 44)	10,000 UI/day (*n* = 41)
Age (years), median (IQR)	65 (53–74)	64 (44–72)	67 (58–75)
Time from symptoms onset to hospital admission (days), media (IQR)	7 (6–10)	7 (6–10)	7 (6–10)
Sex			
Men— no. (%)	60 (71%)	30 (68%)	30 (73%)
Women— no. (%)	25 (29%)	14 (32%)	11 (27%)
Ethnicity			
Spanish— no. (%)	68 (80%)	33 (75%)	35 (85%)
African— no. (%)	1 (1%)	1 (2%)	0 (0%)
Latin-American— no. (%)	14 (16%)	8 (18%)	6 (15%)
Asian— no. (%)	1 (1%)	1 (2%)	0 (0%)
Arabic— no. (%)	1 (1%)	1 (2%)	0 (0%)
BMI			
Baseline BMI, mean (SD)—kg/m^2^	30.2 (4.6)	30.6 (4.8)	29.7 (4.3)
Classification by BMI			
Normal Weight (18-5–24.99) — no. (%)	7 (8%)	4 (9%)	3 (7%)
Overweight (25–25.9) — no. (%)	32 (38%)	15 (34%)	17 (41%)
Obesity (>30) — no. (%)	46 (54%)	25 (57%)	21 (51%)
Tobacco use			
Former— no. (%)	25 (29%)	13 (30%)	12 (29%)
Consumer— no. (%)	3 (4%)	1 (2%)	2 (5%)
Alcohol consumption			
Former— no. (%)	4 (5%)	2 (5%)	2 (5%)
Consumer— no. (%)	9 (11%)	4 (9%)	5 (12%)
Coexisting conditions			
Hypertension— no. (%)	41 (48%)	18 (41%)	23 (56%)
Dyslipidaemia— no. (%)	31 (36%)	12 (27%)	19 (46%)
Diabetes— no. (%)	19 (22%)	8 (18%)	11 (27%)
Pneumonia at Rx			
Unilateral— no. (%)	12 (14%)	7 (16%)	5 (12%)
Bilateral— no. (%)	73 (86%)	37 (84%)	36 (88%)
Ventilatory support			
Nasal glasses— no. (%)	72 (85%)	37 (84%)	35 (85%)
Reservoir— no. (%)	13 (15%)	7 (16%)	6 (15%)
Blood biochemistry data			
Vitamin D, mean (SD)—ng/mL	14.8 (6.2)	14.3 (6.2)	15.3 (6.3)
Calcium, mean (SD)— mg/dL	8.7 (0.4)	8.7 (0.4)	8.7 (0.5)
Phosphate, mean (SD)— mg/dL	3.2 (0.6)	3.2 (0.6)	3.4 (0.7)
Treatment			
Dexamethasone— no. (%)	58 (62%)	29 (66%)	29 (71%)
Methylprednisolone— no. (%)	21 (25%)	10 (23%)	11 (27%)
Tocilizumab— no. (%)	21 (25%)	9 (20%)	12 (29%)
Remdesivir— no. (%)	13 (15%)	9 (20%)	4 (10%)
Heparin— no. (%)	73 (86%)	37 (84%)	36 (88%)
Ceftriaxone— no. (%)	50 (59%)	24 (55%)	26 (63%)
Azithromycin— no. (%)	37 (44%)	20 (45%)	17 (41%)

IQR, interquartile range; SD, standard deviation; no., number. There were no differences between treatment groups.

### Vitamin D Serum Levels

**FIGURE 2:**
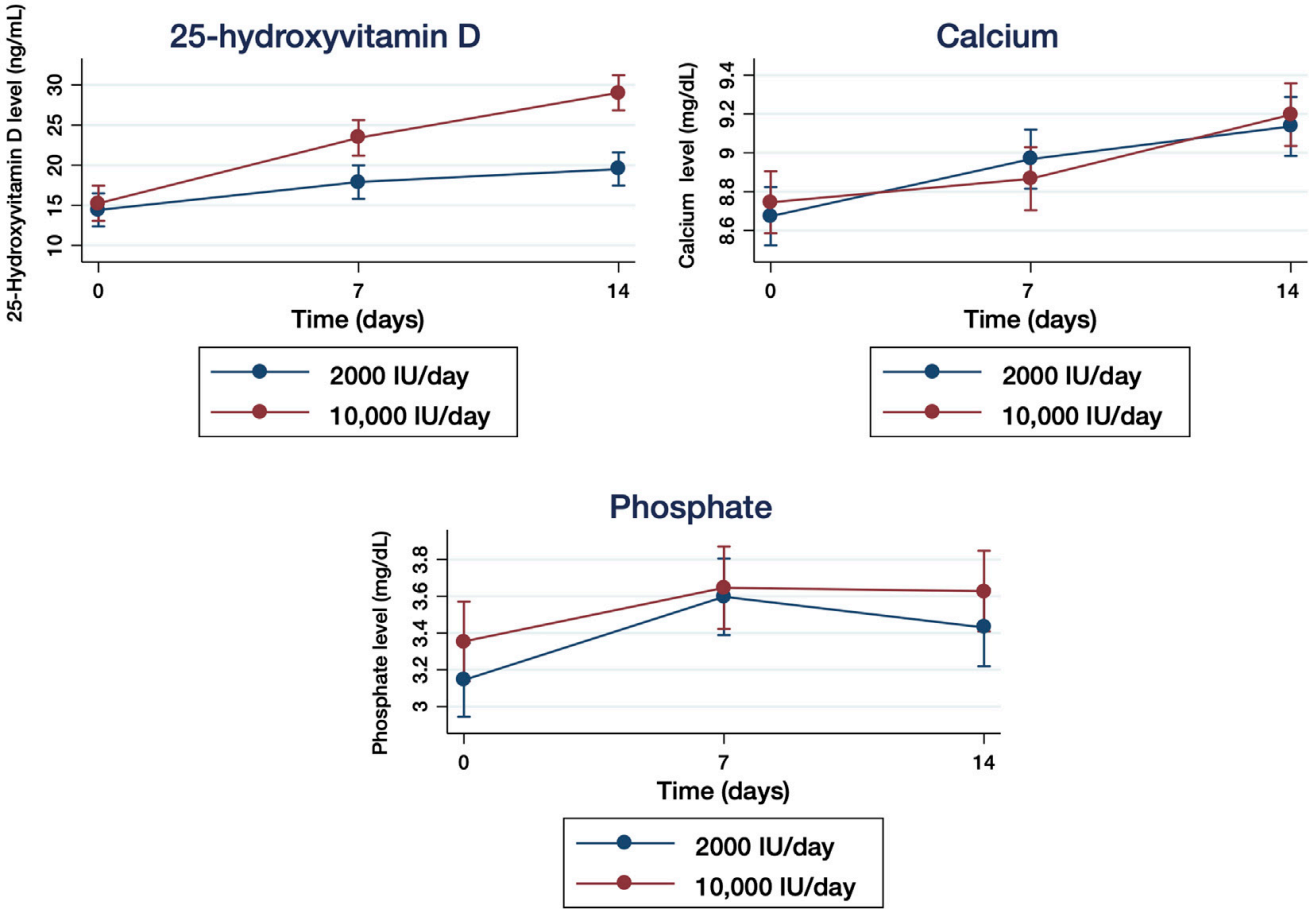
Quantification of serum 25-hydroxyvitamin D (25(OH)D) levels (mg/ml) **(A)**, calcium level (mg/dl) **(B)**, and phosphate level (mg/dl) **(C)** in hospitalized patients with COVID-19 who received 2000 IU/day and 10,000IU/day of cholecalciferol measured at baseline (day 0) and after 7 and 14 days of treatment. The results are presented as means ±95% of confidence intervals.

At the entry in the study, mean serum 25(OH)D levels were very similar in both treatment group (Table 1). After supplementation, target serum 25(OH)D level (≥30 ng/ml) was reached by 7 (16%) participants in the moderate dose group and 20 (49%) in the high dose group (*p* = 0.0021). The mixed-design ANOVA showed a statistically significant interaction between measurement times and treatment groups: F_2.162_ = 25.02, *p* < 0.001 and time: F_2.162_ = 106.21, *p* < 0.001 in the serum 25(OH)D levels (Figure 2A).

### Calcium and Phosphate Serum Levels

Basal calcium levels were 8.7 mg/dl (SD: 0.4) in the moderate dose group and 8.7 mg/dl (SD: 0.5) in the high dose group (Table 1). Basal phosphate levels were 3.2 mg/dl (SD: 0.6) in the 2000 IU/day group and 3.4 mg/dl (SD: 0.7) in the 10,000 IU/day group (Table 1). The mixed-design ANOVA did not identify statistically significant interaction between measurement times and treatment groups in serum calcium: F_2.160_ = 1.52, *p* = 0.22 (Figure 2B) or phosphate: F_2.158_ = 0.24, *p* = 0.79 (Figure 2C) levels, respectively. No episodes of hypercalcemia, hypercalciuria or hyperphosphatemia occurred among the participants in either group as a consequence of the treatment with cholecalciferol.

### Length of the Hospital Stay and ARDS Development

LOS was similar in both groups, 7 days (IQR: 4–9) among the participants who received 2000 IU/day and 7 days (IQR: 4–8) in those who received 10,000 IU/day, without significant differences between groups (Table 2). Multivariable analysis of predictors associated with the LOS is shown in Table 3. We identified that only supplementation with 10,000 IU/day of vitamin D *versus* 2000 IU/day (*p* = 0.01) was associated with a decrease in the LOS, while factors such as age (*p* < 0.001), male gender (*p* = 0.01), high oxygen needs at admission (*p* = 0.001), development of ARDS (*p* < 0.001) and the treatment with corticosteroids (*p* = 0.002) were correlated with a significant increase in the LOS. During the study, 10 (12%) participants developed ARDS. Of them, 6 (14%) participants had been randomly assigned to the 2000 IU/day group and 4 (10%) patients to the 10,000 IU/day group (Table 2). The analysis of the predictors related to the development of ARDS showed that female gender (*p* = 0.049), high oxygen requirements on admission (*p* = 0.001) and D-dimer levels >0.5 μg/ml at entry (*p* = 0.047) were associated with significant risk (Table 4). According to ARDS, LOS was significantly different in the groups of vitamin D_3_ supplementation. Those patients who received 2000 IU/day and developed ARDS stayed at the hospital a median of 27 days (IQR: 12–45), whereas the participants who received 10,000 IU/day stayed 7 days (IQR: 4–13) (*p* = 0.04) (Table 5). In those participants who developed ARDS, the analysis of the needs for non-invasive mechanical ventilation (NIV) between the groups was not significantly different (*n* = 3 in 2000 IU/day group vs*. n* = 1 in 10,000 IU/day group). Similarly, there were no significant differences among these participants in the ICU admission rate (*n* = 5 in the 2000 IU/day group vs*. n* = 1 in the 10,000 IU/day group) (Table 5).

**TABLE 2:** Clinical characteristics in hospitalized patients with COVID-19 who received 2000 IU/day and 10,000IU/day of cholecalciferol.

Characteristics	Overall	Treatment Group	
	(*n* = 85)	2000 UI/day (*n* = 44)	10,000 UI/day (*n* = 41)
Length of hospital stay (days), median (IQR)	7 (4–8)	7 (4–9)	7 (4–8)
ARDS— no. (%)	10 (12%)	6 (14%)	4 (10%)
Admission to ICU— no. (%)	6 (7%)	5 (11%)	1 (2%)
In-hospital mortality— no. (%)	2 (2%)	1 (2%)	1 (2%)

ARDS, acute respiratory distress syndrome; ICU, intensive care unit; IQR, interquartile range; no., number. There were no differences between treatment groups.

**TABLE 3:** Multivariable analysis predicting factors associated with the length of hospital stay (LOS) in hospitalized patients with COVID-19.

Variables	Multivariable		
	β Coefficient	95% CI	*p*-value
Age	0.02	0.01–0.02	**<0.001**
Male gender	0.3	0.06–0.58	**0.01**
Radiologic score	−0.04	−0.12–0.02	0.2
Oxygen flow at entry	0.06	0.03–0.09	**0.001**
ARDS	0.8	0.4–1.2	**<0.001**
Vitamin D; 10,000 UI/day dose	−0.3	−0.6–-0.065	**0.01**
Dexamethasone	0.6	0.2–1.0	**0.002**
Methylprednisolone	0.7	0.2–1.1	**0.002**
Tocilizumab	0.2	−0.062–0.5	**0.1**

ARDS, acute respiratory distress syndrome; CPR, C reactive protein; LDH, lactate dehydrogenase. 95% CI, 95% confidence interval. The variable LOS was transformed into a natural logarithm to adjust to a linear model. Statistical significance is indicated in bold.

**TABLE 4:** Predictive factors related to acute respiratory distress syndrome (ARDS) in hospitalized patients with COVID-19.

Variables	Multivariable		
	OR	95% CI	*p*-value
Female	6.1	1.0–37.0	**0.049**
Oxygen flow at entry	1.5	1.2–1.8	**0.001**
Basal LDH, >225 U/L	0.1	0.007–2.668	0.1
Basal D-Dimer, >0.5 μg/ml	0.98	0.976–0.999	0.047

CPR, C reactive protein; LDH, lactate dehydrogenase; OR, odds ratio; 95% CI, 95% confidence interval. Statistical significance is indicated in bold.

**TABLE 5:** Clinical characteristics in hospitalized patients with COVID-19 who developed acute respiratory distress syndrome (ARDS) during treatment with 2000 IU/day or 10,000IU/day of cholecalciferol.

Clinical Characteristics	Treatment Group		*p*-value
	2000 UI/day (n = 6)	10,000 UI/day (n = 4)	
Length of hospital stay (days), median (IQR)	27 (12–45)	7 (4–13)	**0.04**
Non-invasive mechanical ventilation— no. (%)	3 (50%)	1 (25%)	0.57
ICU admisssion — no. (%)	5 (83%)	1 (25%)	0.19

ICU, intensive care unit; IQR, interquartile range; no., number. Statistical significance is indicated in bold.

### Oxygen Supplementation

At the beginning of the study, all participants required supplemental oxygen. Of them, 37 (84%) participants in the 2000 IU/day group and 35 (85%) participants in the 10,000 IU/day needed nasal prongs, whereas 7 (16%) participants in the 2000 IU/day group and 6 (15%) in the 10,000 IU/day group needed reservoir masks (Table 1). During the study, the supplemental oxygen therapy varied from NIV, high-flow nasal cannulae, reservoir masks, or nasal goggles to participants without oxygen requirements, although there were no significant differences between the groups after 7 or 14 days (Table 6). The analysis of the oxygen requirements after 14 days, measured by FiO_2,_ was significantly reduced 7.6-fold in the high dose group compared to the moderate dose group (*p* = 0.049).

**TABLE 6:** Oxygen therapies in hospitalized patients with COVID-19 after 7 and 14 days of treatment with 2000 IU/day or 10,000IU/day of cholecalciferol.

Ventilatory Support	Treatment Group				Between groups p-value^a^	Between groups p-value^b^
	2000 UI/day (*n* = 44)		10,000 UI/day (*n* = 41)			
	7 days	14 days	7 days	14 days		
No oxygen — no. (%)	35 (80%)	39 (89%)	30 (73%)	40 (98%)	0.77	0.22
Nasal glasses — no. (%)	5 (11%)	1 (2%)	8 (20%)	0 (0%)		
Reservoir — no. (%)	2 (5%)	0 (0%)	1 (2%)	1 (2%)		
High-flow nasal cannulae — no. (%)	2 (5%)	1 (2%)	1 (2%)	0 (0%)		
Non-invasive mechanical ventilation — no. (%)	0 (0%)	3 (7%)	0 (0%)	0 (0%)		

a After 7 days.

b After 14 days.

### Body Mass Index

**FIGURE 3:**
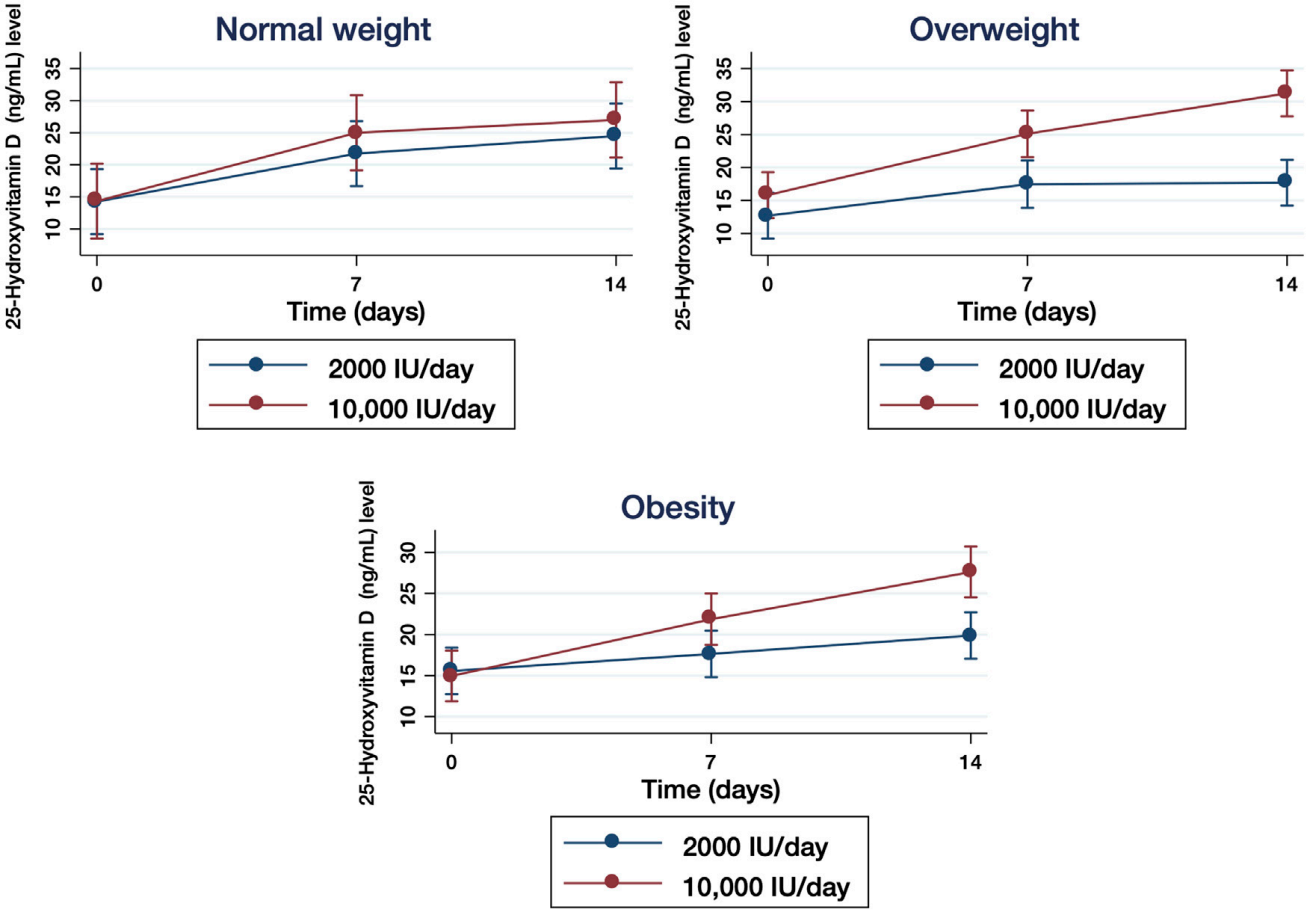
Quantification of serum 25-hydroxyvitamin D (25(OH)D) levels in hospitalized patients with COVID-19 who received 2000 IU/day and 10,000IU/day of cholecalciferol classified by the body mass index in normal-weight (BMI:18-5–24.99), overweight (BMI: 25.0–29.9) and obese (BMI ≥30) participants measured at baseline (day 0) and after 7 and 14 days of treatment. The results are presented as means ±95% of confidence intervals.

In the group of participants who received 2000 IU/day, mean BMI was 30.6 (SD: 4.8) and overweight (OW) was identified in 40 patients, of whom 25 (57%) were obese. Among the participants who received 10,000 IU/day, mean BMI was 29.7 (SD: 4.3) and OW was found in 38 participants, being 21 (51%) classified as obese (Table 1). After 7 days of supplementation, there was a non-significant trend between the moderate dose group and the high dose group both normal-weight (Figure 3A) and obese (Figure 3C) participants. In contrast, the vitamin D levels of OW participants in the high dose group were significantly higher compared to participants of the moderate group (*p* = 0.005) (Figure 3B). At the end of supplementation (14 days), serum vitamin D levels in normal-weight participants remained with a non-significant trend between treatment groups (Figure 3A) and significantly increased in OW (*p* < 0.0001) and obese (*p* < 0.0001) participants (Figures 3B,C). The mixed-design ANOVA showed a statistically significant interaction between measurement times and treatment groups: F_2.162_ = 25.02, *p* < 0.001 and time: F_2.162_ = 106.21, *p* < 0.001 in the serum 25(OH)D levels (Figure 2A).

### Serological Parameters

**FIGURE 4:**
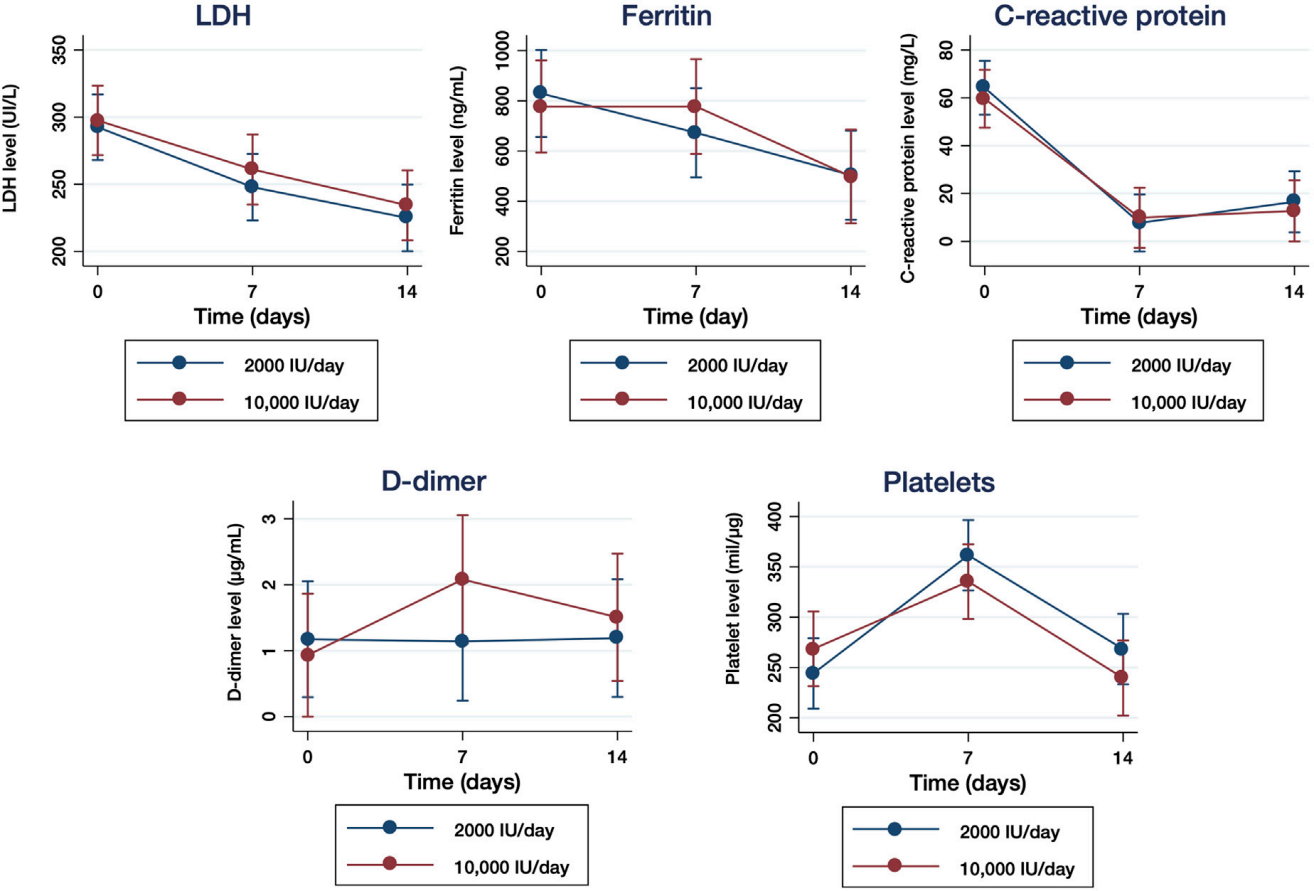
Serological biomarkers related to the inflammation in samples of hospitalized patients with COVID-19 who received 2000 IU/day and 10,000IU/day of cholecalciferol. Levels (U/L) of lactate dehydrogenase (LDH) **(A)**, levels (ng/ml) of ferritin **(B)**, levels (mg/L) of C-reactive protein [CRP] **(C)**, levels (µL/ml) of D-Dimer **(D)** and platelet count (mil/µl) **(E)** were quantified at baseline (day 0), 7 and 14 days of treatment. The results are presented as means ±95% of confidence intervals.

We analyzed the serological biomarkers related to inflammation and considered them as prognostic factors for COVID-19 severity. LDH levels decreased significantly 1.18- (*p* = 0.0023) and 1.29- (*p* < 0.0001) fold in the moderate group after 7 and 14 days, respectively (Figure 4A). Similarly, in the high dose group, LDH levels were significantly reduced 1.23-fold (*p* < 0.0001) and 1.34-fold (*p* < 0.0001) after 7 and 14 days, respectively. Ferritin levels also showed a significant reduction of 1.39-fold after 7 days of treatment (*p* = 0.0013) and 1.96-fold (*p* < 0.0001) after 14 days of treatment in the moderate dose group, whereas it was reduced 1.15-fold (*p* = 0.0033) and 1.66-fold (*p* < 0.0001) in the high dose group after 7 and 14 days of treatment, respectively (Figure 4B).

CRP levels were significantly reduced 8.20-fold (*p* < 0.0001) after 7 days and 8.34-fold (*p* < 0.0001) after 14 days in the 2000 IU/day group, whereas they were reduced 7.47- (*p* < 0.0001) and 6.96- (*p* < 0.0001) fold in the 10,000 IU/day group after 7 and 14 days, respectively (Figure 4C). D-Dimer levels remained unchanged in the moderate dose group after 7 days of treatment and increased slightly after 14 days, but without statistical significance, whereas in the high dose group D-dimer remained unchanged after 7 and 14 days (Figure 4D). In the case of platelet count, the levels detected increased significantly after 7 days of treatment (*p* < 0.0001) in both groups, but decreased to basal levels in both moderate and high dose groups after 14 days of treatment, without statistical significance (Figure 4E). The analysis of the comparisons over time did not show significant differences in LDH, ferritin, CPR or D-dimer serum levels, although it was significantly different for the platelet count (*p* = 0.049).

### Safety

Thirteen different adverse events were documented in the study participants during the study: 7 adverse events occurred in the 2000 IU/day group and 8 in the 10,000 IU/day group, although none of them was directly attributed to the administration of cholecalciferol (Table 7). The total number of participants experiencing adverse events was 17 in both groups, being 9 (20%) in the moderate dose group and 8 (20%) in the high dose group, without statistically significant differences between groups. The most frequent adverse events recorded were pulmonary embolism (n = 3; 4%) and urinary tract infections (n = 3; 4%), which were diagnosed after hospital discharge.

**TABLE 7:** Adverse events in hospitalized patients with COVID-19 who received 2000 IU/day and 10,000IU/day of cholecalciferol.

Adverse Events	Treatment Group	
	2000 UI/day (*n* = 44)	10,000 UI/day (*n* = 41)
Cytolysis— no. (%)	1 (2%)	
Diarrhea— no. (%)	1 (2%)	
Pulmonary embolism— no. (%)	2 (5%)	1 (2%)
Neuropathy— no. (%)	1 (2%)	
Urinary (tract) infection— no. (%)	2 (5%)	1 (2%)
Thyroiditis— no. (%)	1 (2%)	
Hypertriglyceridemia— no. (%)	1 (2%)	
Bacteremia— no. (%)		1 (2%)
Diabetes— no. (%)		1 (2%)
Renal infarction— no. (%)		1 (2%)
Bacterial pneumonia— no. (%)		1 (2%)
Tachycardia— no. (%)		1 (2%)
Thrombocytopenia— no. (%)		1 (2%)

## Discussion

Vitamin D is a fat-soluble secosteroid hormone with a well-defined spectrum of immunomodulatory, anti-inflammatory, and antioxidant actions (Ebadi and Montano-Loza, 2020). Its deficiency is considered a public health issue that has been identified in over half of the population worldwide in people of all age groups (Rubin, 2021). Vitamin D deficiency has been associated not only with non-communicable diseases but also with increased susceptibility to infectious diseases; especially, upper respiratory tract infections (RTI) (Cannell et al., 2006; Ginde et al., 2009) and higher risk of community-acquired pneumonia (Zdrenghea et al., 2017; Zhou et al., 2019). Similarly, there is now sufficient evidence to support that vitamin D deficiency may predispose to SARS-CoV-2 infection and increase COVID-19 severity and mortality (Rhodes et al., 2021; Seal et al., 2022; Wang et al., 2022) due to several mechanisms: first, vitamin D reduces lung permeability by modulating the renin-angiotensin system (RAS) pathway and angiotensin converting enzyme 2 (ACE2) expression; second, it promotes the production of antimicrobial peptides in the epithelium of the respiratory tract, thereby reducing the possibility of virus infection and COVID-19 symptoms; third, vitamin D favours an anti-inflammatory environment, mostly by reducing the concentration of pro-inflammatory cytokines such as tumor necrosis factor (TNF)-α and IFNγ and by increasing the expression of anti-inflammatory cytokines by macrophages; and finally, vitamin D stabilizes the physical barriers by contributing to the maintenance of functional tight junctions, gap junctions and adherence junctions (Pedersen et al., 2009; Baeke et al., 2010). On the other hand, COVID-19 is characterized by the dysregulation of the inflammatory response, especially the RAS pathway. Consequently, the degree of immune overactivation has been associated with a poorer prognosis (Hu et al., 2021). Accordingly, the use of anti-inflammatory drugs during COVID-19 has been proved to be beneficial, as occurs with corticosteroids (Recovery Collaborative Group et al., 2021).

Vitamin D deficiency has been linked to geographical differences in ARDS and COVID-19 mortality (Marik et al., 2020). Therefore, vitamin D_3_ supplementation could be used prophylactically to reduce the severity of COVID-19, especially in those regions where hypovitaminosis D is frequent (Panarese and Shahini, 2020). Despite Spain is a country with an adequate amount of daily sunlight, the overall vitamin D intake is lower than the recommended levels of 10 µg/day and consequently, the prevalence of deficient 25(OH)D levels among the Spanish population is high (González-Rodríguez et al., 2013; Olza et al., 2017). Indeed, 78.8% of the participants recruited for our study showed basal 25(OH)D serum levels <20 ng/ml (50 nmol/L). A similar study that was performed in adults who were hospitalized for mild to moderate COVID-19 and were supplemented with a 5,000 IU/day dose of vitamin D_3_ during the same time (14 days), serum 25(OH)D levels raised to sufficiency (≥30 ng/ml) (Sabico et al., 2021). However, these individuals were younger than our cohort and showed median baseline serum 25(OH)D levels that were substantially higher. Accordingly, we decided to provide a higher dose of cholecalciferol (10,000 IU) that was administered daily due to the protective effects are expected to be higher when the individuals received daily or weekly doses versus high dose boluses (Martineau et al., 2019). After the daily supplementation of 10,000 IU cholecalciferol for 14 days, 93% of the participants achieved an increase of serum 25(OH)D levels ≥20 ng/ml versus 32% of the individuals who received the 2000 IU/day dose. Although there was a significant 1.5-fold increase of serum 25(OH)D levels that were close to sufficiency in those participants who received the higher dose of cholecalciferol (29 ng/ml; IQR: 25–34) in comparison to those participants that received the moderate dose (17 ng/ml; IQR: 14–23), the primary outcome of a serum 25(OH)D concentration ≥30 ng/ml after 14 days of treatment was not achieved. This could be due to 25 [OH]D is a negative component of the acute phase response (Waldron et al., 2013; Silva and Furlanetto, 2015) and it is usually reduced during acute inflammatory procedures such as COVID-19 (Antonelli and Kushner, 2021). In fact, the deficiency of vitamin D at hospital admission in individuals with COVID-19 has been appointed as a potential prognostic marker of disease severity that would indicate the progression to the inflammatory form of the disease (Chiodini et al., 2021; Seal et al., 2022). Accordingly, the achievement of the primary endpoint of >30 ng/ml of 25 [OH]D in individuals with COVID-19 may be impaired due to the inflammatory process, even with the higher dose of 10,000 IU/day. However, we determined that the administration of the higher dose of 10,000 IU/day was safe and well tolerated by all the participants in the study as we did not observe episodes of hypercalcemia, hypercalciuria, or hyperphosphatemia that are considered to be the initial signs of vitamin D intoxication, or any other related adverse events.

Previous data from other intervention trials showed a preventive effect of vitamin D_3_ supplementation in acute RTIs that was increased in patients with vitamin D deficiency (Martineau et al., 2017; Jolliffe et al., 2021). However, in individuals hospitalized with COVID-19, the clinical effects of the use of vitamin D_3_ supplementation have yielded mixed results. A Spanish population-based study concluded that patients on cholecalciferol treatment had a lower risk of SARS-CoV2 infection, lower risk of severe COVID-19 and lower COVID-19 mortality than unsupplemented 25(OH)D-deficient patients (Oristrell et al., 2022), and a case-series study in COVID-19 patients who received 50,000 IU/day for 5 days showed a reduction in the inflammatory markers and time for recovery in comparison to those who received 1,000 IU/day (Ohaegbulam et al., 2020). Similarly, a previous RCT in Spain demonstrated that the administration of a high dose (0.532 mg) of calcifediol (25(OH)D_3_) significantly reduced the need for ICU admission (Entrenas Castillo et al., 2020), and a placebo-controlled, double-blinded clinical trial reported that in 106 hospitalized patients, those who received vitamin D had a lower trend for hospitalization, ICU duration, need for ventilator assistance and mortality (Maghbooli et al., 2021). On the contrary, data from previous studies and a systematic review evaluating vitamin D_3_ supplementation found low to moderate certainty evidence that a single high dose of vitamin D_3_ is effective to reduce mortality, LOS, ICU admissions, and D-dimer or CRP levels (da Rocha et al., 2021; Murai et al., 2021). Our results did not report a reduction in the LOS or the ICU admissions when the administration of a high dose of vitamin D_3_ for 14 days was compared with the administration of a moderate dose. However, the oxygen requirements measured by FiO2 were significantly reduced in those patients assigned to the high dose group, thereby confirming previous reports (Ohaegbulam et al., 2020). Interestingly, those participants with ARDS from our cohort who received the higher dose of vitamin D_3_ showed a significant reduction in the LOS of 23.5 days on average in comparison with those patients who received the moderate dose. Despite the small number of patients who accelerated their recovery, these results achieved statistical significance and they were in accordance with previous studies that support that a higher vitamin D_3_ status is associated with a significant reduction in infectivity, morbidity and mortality from SARS-CoV-2 infection (Seal et al., 2022; Wang et al., 2022) and also that treatment with vitamin D_3_ during COVID-19 may also shorten the hospital stay and decrease mortality (Gönen et al., 2021). Moreover, according to previous observations in animal models (Yang et al., 2016), the higher dose of vitamin D_3_ in patients with ARDS would increase the expression levels of ACE2, inducing moderate lung changes that might contribute to reduce the symptoms of this syndrome. In our study, the potential positive effect of vitamin D_3_ could be related to the early administration just after hospitalization, as the accelerated improvement of 25(OH)D_3_ levels may develop an anti-inflammatory environment, thereby helping to reduce the length of stay, ICU admission, and mortality in COVID-19 patients (Holick, 2022). Finally, the biochemical inflammatory parameters related to poor prognosis improved mainly during the first week, regardless of the dose of vitamin D administered, as we did not observe significant changes in the serum levels of LDH, ferritin, CRP, or D-dimer serum levels between the two groups. Although the platelet count showed a modest but significant difference in those patients who were supplemented with the higher dose, these changes may not be clinically meaningful.

On the other hand, a high prevalence of vitamin D deficiency has been described in obese and OW patients (Pereira-Santos et al., 2015), likely caused by a dilution into the greater volumes of fat, serum, liver, and muscle that are present in obese individuals (Vranić et al., 2019), although other mechanisms, such as the sequestration of vitamin D by the adipose tissue may not be completely discarded (Wortsman et al., 2000). Due to risk factors such as excess visceral fat or high BMI have been associated with a higher probability to develop more severe forms of COVID-19 (Zhou et al., 2019), a possible link may exists between COVID-19 severity, vitamin D deficiency and BMI. In our study, significant increases in serum 25(OH)D levels were observed among obese and OW participants who received the highest dose in comparison with those who received the moderate dose, while the serum levels in normal weight participants were not different between the groups. However, most of our participants were either OW or obese, as these factors are related to a higher probability to develop severe COVID-19. Therefore, we may not rule out that this imbalance in the BMI between the participants might be affecting the results. Accordingly, the effectiveness of vitamin D_3_ supplementation in these individuals with high BMI to positively influence the clinical recovery from COVID-19 needs to be further analyzed by interventional studies.

This study presents two potential limitations. First, our study was not designed as a double-blinded RCT due to the Pharmacy services of the different hospitals needed to change the commercial medication into non-commercial syringes to be supplied to the participants that had been assigned to each treatment group, which made necessary that both the Pharmacy service and the Researchers knew the treatment group to which the participant had been assigned. Therefore, we cannot rule out that the single-blinded RCT design might have biased the assessment of more subjective parameters (e.g chest X-ray) in favour of those participants who received the high dose. On the other hand, the beneficial effect of vitamin D_3_ supplementation over the reduction of the LOS in individuals who developed ARDS have been described in a small fraction of the participants (10/85), and although these results reached statistical significance, they need to be considered with caution until confirmation in a larger cohort.

In conclusion, the supplementation of a high dose of 10,000 IU/day of cholecalciferol for 14 days in hospitalized patients with COVID-19 was well tolerated and did not cause significant adverse events in comparison with the moderate dose of 2000 IU/day. The higher dose was more effective than the moderate dose to induce a significant increase of 25(OH)D levels in serum, especially in OW and obese individuals, although the participants did not achieve the target level (≥30 ng/ml). Moreover, the results obtained in our cohort supported that a higher dose of vitamin D_3_ supplementation in association with the standard clinical care may be effective to improve the oxygen requirements during hospitalization by COVID-19 and to reduce the LOS in those individuals who developed ARDS. Although this study supports the notion that vitamin D_3_ supplementation during COVID-19 may be safe, tolerable and beneficial, more randomized clinical trials are needed to determine the true efficacy of vitamin D supplementation as part of the therapeutic arsenal used against COVID-19 disease.

## Members of the Multidisciplinary Group of Study of COVID-19 (MGS-COVID)

David Alonso-Menchén (Hospital Universitario Príncipe de Asturias), Adriana Álvarez Jusdado (Hospital Universitario Severo Ochoa), Daniel Barranco Maroto (Hospital Universitario Severo Ochoa), Ytsel Bello Pastor (Hospital Universitario Severo Ochoa), Cristina Córdoba Chicote (Hospital Universitario Severo Ochoa), Guillermo Cuevas Tascón (Hospital Universitario Infanta Leonor), Víctor Díez Viñas (Hospital Universitario Infanta Leonor), Inés Dorado Manzanares (Hospital Universitario Severo Ochoa), Ismael Escobar Rodríguez (Hospital Universitario Infanta Leonor), Eva Fonseca Aizpuri (Hospital Universitario de Cabueñes), Rosa María Galán Zuheros (Hospital Universitario Severo Ochoa), Concepción García Lacalle (Hospital Universitario Severo Ochoa), Mario García Peña (Hospital Universitario Severo Ochoa), Sebastiano Garotti (Hospital Universitario Severo Ochoa), Marta Gómez Sanz (Hospital Universitario Severo Ochoa), Tamara Gómez Zuil (Hospital Universitario Severo Ochoa), María González Pozuelo (Hospital Universitario Infanta Leonor), Cristina Helguera Amezua (Hospital Universitario de Cabueñes), María del Carmen Herrero Alonso (Hospital Universitario Fundación Alcorcón), Javier Marcos Arias (Hospital Universitario Fundación Alcorcón), Emilio José Martínez Martín (Hospital Universitario Fundación Alcorcón), Javier Montoya Adarraga (Hospital Universitario Infanta Leonor), Patricia Moreira Escrich (Hospital Universitario Severo Ochoa), María Jesús Moro Álvarez (Hospital Universitario Infanta Leonor), Paula Novo González (Hospital Universitario Severo Ochoa), Raquel Paz Peño (Hospital Universitario Severo Ochoa), Alejandro Pérez Cachaldora (Hospital Universitario Severo Ochoa), Miriam Rodríguez Fernández (Hospital Universitario Severo Ochoa), María del Carmen Romero Pérez (Hospital Universitario Severo Ochoa), José Sanz-Moreno (Hospital Universitario Príncipe de Asturias), Mercedes Toral Leiva (Hospital Universitario Severo Ochoa), Nuria Toro Moreno (Hospital Universitario Severo Ochoa), Rafael Torres Perea (Hospital Universitario Severo Ochoa), María Velasco Arribas (Hospital Universitario Fundación Alcorcón), Gema Vico Díaz-Parreño (Hospital Universitario Severo Ochoa), Ana Villanueva Fernández-Ardavín (Hospital Universitario Fundación Alcorcón), Rocío Minaya Carrero (Hospital Universitario Severo Ochoa), Monserrat Rodríguez Miguel (Hospital Universitario Severo Ochoa).

## Data Availability

The raw data supporting the conclusion of this article will be made available by the authors, without undue reservation.
