# Supplementary material for: Beneficial Effect of Short-Term Supplementation of High Dose of Vitamin D3 in Hospitalized Patients With COVID-19: A Multicenter, Single-Blinded, Prospective Randomized Pilot Clinical Trial
Source: Front Pharmacol. 2022 Jul 4;13:863587. doi: 10.3389/fphar.2022.863587 (PMC9289223; doi:10.3389/fphar.2022.863587)
Supplement: Supplementary file 1 [file Table1.DOCX]

**Supplemental Table 1.** Baseline clinical and biochemical characteristics in hospitalized patients with COVID-19 who received 2000 IU/day and 10,000IU/day of cholecalciferol.

| **Characteristics** | **Overall** | **Treatment group** | |
| --- | --- | --- | --- |
|  | **(n=85)** | **2000 UI/day (n=44)** | **10,000 UI/day (n=41)** |
| **Age at diagnosis** (years), median (IQR) | 65 (53-74) | 64 (44-72) | 67 (58-75) |
| **Time from symptoms onset to hospital admission (days),** media (IQR) | 7 (6-10) | 7 (6-10) | 7 (6-10) |
| **Sex** |  |  |  |
| Men— no. (%) | 60 (71%) | 30 (68%) | 30 (73%) |
| Women— no. (%) | 25 (29 %) | 14 (32%) | 11 (27%) |
| **Ethnicity** |  |  |  |
| Spanish— no. (%) | 68 (80%) | 33 (75%) | 35 (85%) |
| African— no. (%) | 1 (1%) | 1 (2%) | 0 (0%) |
| Latin-American— no. (%) | 14 (16%) | 8 (18%) | 6 (15%) |
| Asian— no. (%) | 1 (1%) | 1 (2%) | 0 (0%) |
| Arabic— no. (%) | 1 (1%) | 1 (2%) | 0 (0%) |
| **BMI** |  |  |  |
| Baseline BMI, mean (SD)—kg/m2 | 30.2 (4.6) | 30.6 (4.8) | 29.7 (4.3) |
| **Classification by BMI** |  |  |  |
| Normal Weight (18-5-24.99) — no. (%) | 7 (8%) | 4 (9%) | 3 (7%) |
| Overweight (25-25.9) — no. (%) | 32 (38%) | 15 (34%) | 17 (41%) |
| Obesity (> 30) — no. (%) | 46 (54%) | 25 (57%) | 21 (51%) |
| **Tobacco use** |  |  |  |
| Former— no. (%) | 25 (29%) | 13 (30%) | 12 (29%) |
| Consumer— no. (%) | 3 (4%) | 1 (2%) | 2 (5%) |
| **Alcohol** **consumption** |  |  |  |
| Former— no. (%) | 4 (5%) | 2 (5%) | 2 (5%) |
| Consumer— no. (%) | 9 (11%) | 4 (9%) | 5 (12%) |
| **Coexisting conditions** |  |  |  |
| Hypertension— no. (%) | 41 (48%) | 18 (41%) | 23 (56%) |
| Dyslipidaemia— no. (%) | 31 (36%) | 12 (27%) | 19 (46%) |
| Diabetes— no. (%) | 19 (22%) | 8 (18%) | 11 (27%) |
| **Pneumonia at Rx** |  |  |  |
| Unilateral— no. (%) | 12 (14%) | 7 (16%) | 5 (12%) |
| Bilateral— no. (%) | 73 (86%) | 37 (84%) | 36 (88%) |
| **Ventilatory support** |  |  |  |
| Nasal glasses— no. (%) | 72 (85%) | 37 (84%) | 35 (85%) |
| Reservoir— no. (%) | 13 (15%) | 7 (16%) | 6 (15%) |
| **Score of pneumonia,** mean (SD) | 4.0 (1.7) | 3.9 (1.6) | 4.05 (1.8) |
| **Vital signs** |  |  |  |
| Oxygen flow, median (IQR)— Liters per minute | 2 (2-5) | 2 (2-5) | 2 (2-4) |
| Body temperature, mean (SD)—°C | 36.3 (0.6) | 36.4 (0.7) | 36.2 (0.5) |
| Heart rate, mean (SD)—beats/min | 74 (15) | 73 (16) | 76 (13) |
| Breath rate, mean (SD)—breaths/min | 18 (5) | 19 (6) | 17 (4) |
| Systolic blood pressure, mean (SD)—mm Hg | 129 (18) | 126 (16) | 132 (19) |
| Diastolic blood pressure, mean (SD)—mm Hg | 75 (10) | 74 (9) | 75 (10) |
| **Blood biochemistry data** |  |  |  |
| Leucocytes, mean (SD)— mil/µL | 8.1 (4.3) | 7.3 (3.8) | 8.9 (4.7) |
| Neutrophiles, mean (SD)— mil/µL | 6.3 (4.0) | 5.7 (3.6) | 7.0 (4.4) |
| Lymphocytes, mean (SD)— mil/µL | 1.2 (0.9) | 1.1 (0.5) | 1.4 (1.2) |
| Hemoglobin, mean (SD)— g/dL | 13.8 (1.5) | 13.4 (1.2) | 14.3 (1.7) |
| Platelets, mean (SD)— mil/µL | 256 (111) | 246 (98) | 266 (124) |
| aPTT, mean (SD)— sec | 28.9 (4.6) | 28.9 (3.9) | 28.9 (5.2) |
| PT, mean (SD)— sec | 17.4 (15.7) | 18.5 (18.9) | 16.2 (11.5) |
| Fibrinogen, mean (SD)— mg/dL | 537.2 (160.5) | 547.4 (141.5) | 525 (181) |
| D-dimer, mean (SD)— μg/mL | 1.1 (2.0) | 1.2 (2.4) | 0.9 (1.3) |
| Glucose, mean (SD)— mg/dL | 138.4 (54.9) | 131.7 (48.9) | 146 (60.4) |
| Creatinine, mean (SD)— mg/dL | 0.8 (0.2) | 0.8 (0.2) | 0.8 (0.2) |
| Sodium, mean (SD)— mEq/L | 139.6 (3.3) | 139.5 (3.5) | 139.6 (3.2) |
| Potassium, mean (SD)— mEq/L | 4.4 (0.4) | 4.4 (0.4) | 4.5 (0.5) |
| Albumin, mean (SD)— g/dL | 4.1 (3.8) | 4.6 (5.3) | 3.7 (0.5) |
| Bilirubin, mean (SD)— mg/dL | 0.4 (0.2) | 0.4 (0.1) | 0.5 (0.3) |
| LDH, mean (SD)—U/L | 295 (91.2) | 292 (91.7) | 298 (91.7) |
| ALT/GPT, mean (SD)—U/L | 66.8 (90.0) | 75.3 (106.1) | 57.7 (68.9) |
| AST/GOT, mean (SD)—U/L | 44 (34) | 51 (41) | 37.1 (24.2) |
| C-reactive protein, mean (SD)—mg/L | 62.1 (55.1) | 65.2 (55.2) | 58.6 (55.4) |
| Glomerular Filtration, mean (SD)—ml/min/1.73m2 | 91.3 (17.8) | 92.7 (19.6) | 89.9 (15.8) |
| Ferritin, mean (SD)—ng/mL | 804.8 (630.9) | 784.3 (656.2) | 826.8 (610.0) |
| Procalcitonin, mean (SD)—ng/mL | 0.2 (0.3) | 0.1 (0.2) | 0.2 (0.4) |
| PTHi, mean (SD)—pg/mL | 64 (34) | 67 (35) | 61 (34) |
| **Symptoms** |  |  |  |
| Fever— no. (%) | 72 (84.7) | 38 (86.4) | 34 (82.9) |
| Malaise— no. (%) | 80 (94.2) | 40 (90.1) | 40 (97.6) |
| Upper respiratory tract symptoms— no. (%) | 52 (61.2) | 32 (72.7) | 20 (48.8) |
| Dyspnea— no. (%) | 56 (65.9) | 30 (68.2) | 26 (63.4) |
| Chest pain— no. (%) | 24 (28.2) | 14 (31.2) | 10 (24.4) |
| Cough— no. (%) | 64 (75.3) | 35 (79.5) | 29 (70.7) |
| Expectoration— no. (%) | 18 (21.2) | 7 (15.9) | 11 (26.8) |
| Hemoptysis— no. (%) | 1 (1.2) | 0 (0) | 1 (2.4) |
| Myalgia— no. (%) | 48 (56.5) | 25 (56.8) | 23 (56.1) |
| Headache— no. (%) | 21 (24.7) | 10 (22.7) | 11 (26.8) |
| Confusion— no. (%) | 6 (7.1) | 2 (4.5) | 4 (9.75) |
| Seizures— no. (%) | 2 (2.3) | 0 (0) | 2 (4.9) |
| Abdominal pain— no. (%) | 10 (11.2) | 3 (6.8) | 7 (17.1) |
| Nausea/Vomiting— no. (%) | 10 (11.2) | 5 (11.4) | 5 (26.8) |
| Diarrhea— no. (%) | 18 (21.2) | 8 (18.2) | 10 (24.4) |
| Rash— no. (%) | 4 (4.7) | 2 (4.5) | 2 (4.9) |
| Anosmia— no. (%) | 20 (23.5) | 10 (22.7) | 10 (24.4) |
| **Treatment** |  |  |  |
| Dexamethasone— no. (%) | 58 (62%) | 29 (66%) | 29 (71%) |
| Methylprednisolone— no. (%) | 21 (25%) | 10 (23%) | 11 (27%) |
| Tocilizumab— no. (%) | 21 (25%) | 9 (20%) | 12 (29%) |
| Remdesivir— no. (%) | 13 (15%) | 9 (20%) | 4 (10%) |
| Heparin— no. (%) | 73 (86%) | 37 (84%) | 36 (88%) |
| Ceftriaxone— no. (%) | 50 (59%) | 24 (55%) | 26 (63%) |
| Azithromycin— no. (%) | 37 (44%) | 20 (45%) | 17 (41%) |

ALT/GPT, alanine aminotransferase; aPTT, activated partial thromboplastin time; AST/GOT, aspartate aminotransferase; BMI, body mass index; HFNC, high flow nasal cannula; IQR, interquartile range; LDH, lactate dehydrogenase; no., number; PT, prothrombin time; PTHi: intact parathyroid hormone; SD, standard deviation; yr, year. There were no differences between groups regarding the demographic characteristics, symptoms, vital signs, blood biochemistry data, or treatments, although the level of haemoglobin was significantly higher (p=0.006) in the group of participants assigned to the 10,000 IU/day group.
